# Seroprevalence of rubella in pregnant women in Southern Morocco

**DOI:** 10.11604/pamj.supp.2020.35.1.18496

**Published:** 2020-02-10

**Authors:** Hanane Zahir, Lamiae Arsalane, Ghita Elghouat, Hanane Mouhib, Youssef Elkamouni, Said Zouhair

**Affiliations:** 1 Laboratoire de Microbiologie-Virologie et Biologie Moléculaire, Hôpital Militaire Avicenne, Centre Hospitalier Universitaire Mohammed VI de Marrakech, Faculté de Médecine et de Pharmacie de Marrakech, Université Cadi Ayyad, avenue Ibn Sina, BP 2360 Marrakech-principal, Maroc

**Keywords:** Pregnant women, rubella, seroprevalence

## Abstract

Rubella is a generally benign but dangerous viral infection in early pregnancy, due to the teratogenic potential of the virus. Indeed, it causes spontaneous abortions, in-utero fetal death, premature labor and congenital malformations known as congenital rubella syndrome. The purpose of this study is to determine the immune status of rubella in pregnant women in southern Morocco. A prospective, multicentre study was conducted in 2017 for the detection of rubella IgG and IgM antibodies in 380 pregnant women aged 17 to 46 years, using the Architect i1000 chemiluminescent microparticle immunoassay. Eigthy for percent (84.7%) of women were seropositive. Ten percent of multiparous women remained seronegative despite recommendations for vaccination after delivery. Preventive measures against congenital rubella need to be strengthened, and vaccination is needed in non-immunized women. Vaccination awareness campaigns, especially among non-immunized multiparous women, remain essential.

## Introduction

Rubella is an acute viral disease, basically one of children. Its clinical course is generally favorable in almost all cases when it affects the child in the postnatal period. It is, however, a real public health problem because of the teratogenicity of the virus. When a woman contracts the disease during pregnancy, the consequences can be dramatic for the fetus, especially when the infection takes place in the first trimester, sometimes leading to spontaneous abortion, fetal death, or the birth of a child with congenital malformations known as Congenital rubella syndrome (CRS). Vaccination or, better, early natural infection are the only ways to prevent this important disease [1]. In Morocco, the epidemiology of rubella remains poorly understood, since it is not a reportable disease. This study has for its objectives the determination of the rubella immunity status of pregnant women in southern Morocco and the effort to establish a link between rubella seroprevalence and the socio-demographic factors studied.

## Methods

This is a cross-sectional, multicentre study, both descriptive and analytic, done in 2017 at the Bacteriology and Virology Laboratory of the Avicenna Military Hospital, Marrakech. The study includes 380 pregnant women, either hospitalized or consulting at one of three hospitals: the Hassan II Hospital, Agadir, the Avicenna Military Hospital, Marrakech, and the Ourzazate Provincial Hospital Center, and one of the three Ouarzazate regional health centers. The nature of the study was carefully explained to the study population and oral consent was obtained from each participant. A questionnaire was filled for each woman covering age, socio-demographic factors, gestational age, previous obstetrician-gynecological (OB-GYN) care and vaccinations received. IgG and IgM studies were done with ARCHITECT i1000 (Abbott Diagnostics), closed systems immunoanalysis, based on chemiluminescent microparticle immunoassay. For IgM, a result counts as positive (reactive) if the sample value is > 1 and negative (non-reactive) with a value < 1. For IgG, a result was considered positive if the IgG value was ≥ 10.0 IU/ml, negative if the value was between 0 and 4.9, and ambiguous if between 5.0 and 9.9 IU/ml. In our study, ambiguous values were considered negative. Data entry was done using Excel©, and statistical analysis used SPSS, ver. 19 for Windows. The study of association between rubella sero-immunity and socio-demographic characteristics was based on chi^2^ and Fisher’s exact test for qualitative variables. Values were considered statistically significant at the level of p < 0.05.

## Results

**Figure 1:**
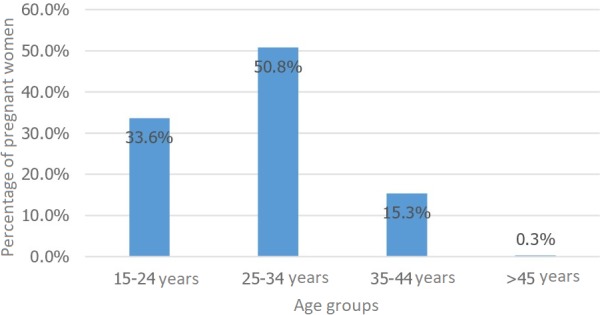
Distribution of patients by age range

**Figure 2:**
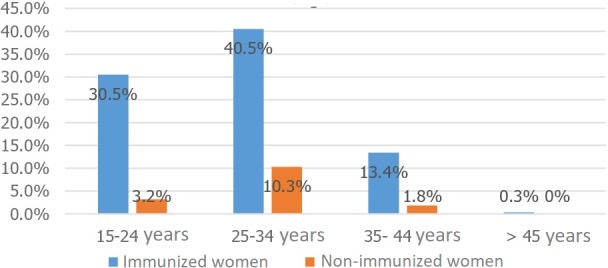
Distribution of immunized and non-immunized women by age range

**Figure 3:**
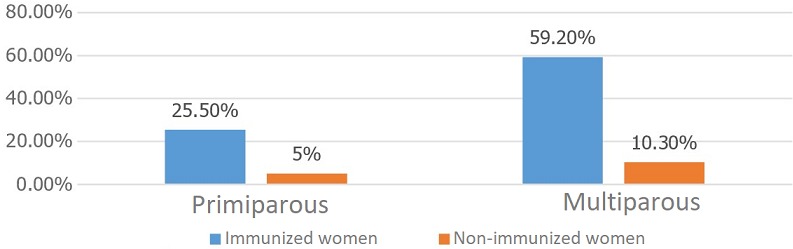
Immune status of pregnant women by parity

The mean age of the patients was 28, ranging from 17 to 46 years. The age range 25-34 was most represented, accounting for 50.8 percent of the cases (Figure 1). Of the women studied, 41 percent were in their third trimester, the remainder divided between first and second trimesters. Of the patients, 69.5 percent were multiparous, 15 percent had had a miscarriage and 4.5 percent had a history of in-utero fetal death. Women of a middle social and educational status were, respectively, 84 and 74 percent of the study population. Among the 380 women studied, 84.7 percent were IgG positive, while all the women were IgM negative (there was no current risk of rubella infection). The age range 25 through 34 was the most likely to have immunity, with seropositivity of 40.5 percent and seronegativity of 10.3 percent (Figure 2). Some 51.5 percent of the immune women lived in urban areas, and 30.5 percent in rural areas. Ten percent (10.3%) of the multiparous women remained seronegative for rubella during their previous pregnancies (Figure 3). There was no statistically significant relationship between rubella immunity and all of the factors cited (p < 0.05).

## Discussion

Rubella is generally a benign viral infection, but can cause spontaneous abortion, fetal death or the birth of a child with congenital malformations when the pregnant woman is exposed to the virus during the first trimester of pregnancy. In case of infection before 12 weeks, the frequency of fetal infection is 90 percent and the risk of major fetal anomalies is very high (on the order of 90 percent) [1].

In Morocco, the epidemiology of rubella remains poorly understood, since it is not a reportable disease. Studies, very limited and done at the national level (Rabat, Meknès), examined IgG antibody seroprevalence among pregnant women. There have been susceptibility reports of 11.3 percent [2] and 9.8 percent [3]. In this study, 84.7 percent of women were seropositive for rubella immunity (antibody value > 10IU/ml). This immunity rate is comparable to those reported at the national level. Other studies in numerous countries have reported seroprevalence from 58 percent to 98 percent [4–11] (Table 1). The significant difference in rubella immune status in different countries can be explained by the date of rubella vaccine introduction, of any mass vaccination campaigns, and of sensitization of the population. According to the results of this study, there is no significant relation between the IgG seropositivity and the various factors studied, notably age, rural/urban origin, parity, and socio-economic and educational levels.

**Table 1:** National and international comparison of rubella seroprevalence

Study	Year	Seropositivity
Our study	2017	84.7%
Meknes (Morocco) [2]	2015	88.7%
Rabat (Morocco) [3]	2009-2011	90.2%
Tunisia [4]	2010	79.7%
Canada [5]	2008-2011	85%
Namibia [6]	2010	85%
China [7]	2010-2012	58.4%
Brazil [8]	2007-2012	97.2%
Spain [9]	2008-2013	94.1%
Norway [10]	2010-2011	94.4%
Togo [11]	2013	85%

Analysis of the qualitative rubella serology results shows that none of the women were IgM positive, and that there had not been any recent infection during the period of the study. Specific IgM can be detected not only in the case of a recent primary infection, but also in the case of a reinfection (a highly exceptional situation), or because of non-specific polyclonal stimulations of the immune system, as well as a cross reaction with rheumatoid factors in the case of systemic disease. Because of these different situations during which IgM is detectable, recourse to complementary tests like IgG avidity is indispensable to confirm or deny a diagnosis of recent infection. The use of this technique rests on the fact that the avidity matures with the time before the start of the infection. Thus, a weak rubella IgG avidity shows a recent infection, while a high avidity permits the exclusion of a recent primary infection [12].

The global coverage of rubella vaccination is on the rise, having gone from 21 percent in 2000 to 40 percent in 2012, then to 47 percent in 2016 [13]. Nonetheless, the Moroccan vaccination program does not take women of child bearing age into consideration. The decline in incidence of the number of CRS cases in Morocco would be possible only if the virus circulation were interrupted by mass vaccination of women of child bearing age, and of school age girls, along with routine vaccination of children with Measles-Rubella (MR) or Measles, Mumps and Rubella (MMR). In our study, 10 percent of the multiparous women were seronegative for rubella in their previous pregnancies, though they should have been immunized [14]. The World Health Organization recommends that every seronegative pregnant woman, or one whose immune status is unknown, should be vaccinated post-partum before hospital discharge in order to achieve a seroprevalence of 100 percent [2]. The non-vaccination of seronegative women is explained by the lack of communication between patients and health personnel about knowing one´s immune status before marriage, and the importance of post-partum vaccination.

## Conclusion

Congenital rubella is a serious condition which should be eradicated, since there is a live attenuated vaccine against the disease. Every women of reproductive age needs to be immunized. Premarital rubella serodiagnosis is recommended, since interpretation of the serology becomes more complicated if done during pregnancy. Despite implementation of rubella vaccination in the national vaccination program, seronegativity remains high when compared to the eradication objectives of the Ministry of Public Health. Vaccine sensitization campaigns, especially among unvaccinated multiparous women, remain indispensable to achievement of the objectives.

### What is known about this topic

- The epidemiology of rubella remains poorly understood, since it is not a reportable disease;
- Vaccination or early natural infection are the only effective ways to prevent this disease.

### What this study adds

- The rate of sero-negativity remains high when compared to the eradication objectives of the Ministry of Public Health;
- Vaccination sensitization campaigns for non-immunized women are indispensable for control of congenital rubella syndrome.

## Competing interests

The authors declare no competing interests.
